# Equitable intelligence for Africa: strengthening global health artificial intelligence (AI) governance

**DOI:** 10.11604/pamj.2025.52.179.50386

**Published:** 2025-12-23

**Authors:** Luchuo Engelbert Bain, Basile Njei

**Affiliations:** 1 The African Population and Health Research Center (APHRC), Nairobi, Kenya,; 2 Department of Psychology, Faculty of Humanities, University of Johannesburg, Auckland Park, Johannesburg, South Africa,; 3 Section of Digestive Diseases, Department of Medicine, Yale University, New Haven, Connecticut, United States,; 4 Engelhardt School of Global Health and Bioethics, Euclid University, Central African Republic,; 5 Artificial Intelligence Programme, University of Cumbria, Carlisle, United Kingdom,; 6 Ohio University Heritage College of Osteopathic Medicine, Athens, Ohio, United States,; 7 Yale Liver Center, Yale New Haven Health, New Haven, Connecticut, United States

**Keywords:** Equity, artificial intelligence, global health

## Editorial

As artificial intelligence (AI) rapidly reshapes global health, one of the most fundamental universal principles, equity, risks being sidelined [1]. Despite decades of debate, the global health community still lacks conceptual clarity and operational consensus on what equity truly means in research, partnerships, and governance. The renewed push for decolonizing global health reflects long-standing concerns about inequitable power dynamics, extractive research practices, and disproportionate agenda-setting by institutions in the global north. However, without deliberate safeguards, the expansion of AI threatens to amplify these disparities on an unprecedented scale.

**Opportunities: how AI can reduce inequities in global health:** artificial intelligence (AI) holds enormous potential to narrow global health inequities. AI can expand access to quality care through low-cost diagnostic tools on smartphones, empower community health workers with decision-support systems, and strengthen population-level surveillance to predict outbreaks earlier and allocate scarce resources more efficiently. AI can also compensate for specialist shortages by supporting image interpretation, triage, and treatment planning in oncology, radiology, and primary care [1,2].

But to harness this potential, Africa must invest in algorithmic literacy, strengthen local capacity to understand how models work, and build the technical talent needed to generate, govern, and interpret its own data. Most importantly, AI becomes an equity tool only when African researchers train models on African data, rooted in local disease patterns, languages, and cultural realities. This will ensure technology serves African needs rather than importing solutions misaligned with context.

**Risks: how AI can widen inequities in global health:** artificial intelligence (AI) can also widen inequities if introduced without ethical and contextual grounding or if poorly governed. Models trained on non-representative datasets, largely from wealthy, Western populations, have the potential to produce biased, harmful outputs when deployed in African settings [2]. For example, skin cancer algorithms trained on predominantly light skin tones perform poorly in validation studies in the African population [3]. Without local oversight, AI risks reinforcing existing power asymmetries, enabling extractive data practices, and locking African countries into technological dependence. When nations deploy AI systems, they neither built nor understand, they relinquish the ability to detect bias, contest decisions, or safeguard patient rights. To better put it, without local expertise to audit AI algorithms, African health systems risk becoming passive recipients of potentially harmful external technology.

Unequal access to costly AI systems may further widen the gap between well-resourced and under-resourced health systems. Africa cannot afford passive adoption. Without deliberate capacity strengthening, including data science training, regulatory frameworks, ethical governance, and continental coordination, AI could amplify digital divides, externalize control of health data, and entrench structural inequities for decades to come.

**Solution: the need for an AI equity index for global health:** equity must be explicitly defined, consistently understood, and rigorously measured. Without a shared framework, global health risks reinforce the very injustices it aims to correct [4]. Clear metrics have the potential to reveal who is being excluded, how structural biases operate, and where corrective action is needed, especially as AI systems can amplify existing disparities. Without shared measures, equity remains rhetoric. AI can advance health equity, but only if we confront the biases embedded in its design, datasets, and deployment.

To prevent AI from reproducing or deepening structural inequities, we argue that it is critical to develop and streamline a comprehensive AI equity index and scale for global health, integrating domains such as: 1) data representation and diversity; 2) algorithmic fairness and bias mitigation; 3) transparency and explainability; 4) governance and oversight; and 5) community benefit sharing and data ownership. Such tools would embed equity metrics into AI design, deployment, and evaluation; strengthen accountability; guide ethical implementation; and provide governments with evidence to justify responsible AI investments. The African context demands these tools urgently.

AI offers unprecedented potential to strengthen health systems, enhance precision in resource allocation, and reduce long-standing inequities in access, quality, and outcomes. However, when trained on prejudiced or exclusionary data, AI amplifies existing injustices. Without rigorous regulation, contextual relevance, and equity-centered oversight, AI risks deepening the very inequalities it claims to solve. Advancing Africa´s position in global health requires an all-of-society commitment to strategic AI investment. Failing to invest risks widening disparities and deepening dependence. Africa cannot afford a reactive posture. AI is no longer merely a technological asset; it is fast becoming a diplomatic currency that will shape negotiation power, agenda-setting, and influence within global health governance. Meaningful AI capacity will determine whether African countries enter future negotiations as policy takers or equitable partners.
